# Triple Silencing of HSP27, cFLIP, and CLU Genes Promotes the Sensitivity of Doxazosin-Induced Apoptosis in PC-3 Prostate Cancer Cells

**DOI:** 10.3390/medicines11030007

**Published:** 2024-02-21

**Authors:** Jeong Man Cho, Sojung Sun, Eunji Im, Hyunwon Yang, Tag Keun Yoo

**Affiliations:** 1 Department of Urology, Uijeongbu Eulji Medical Center, Uijeongbu 11759, Republic of Korea; uro02@eulji.ac.kr; 2 Department of Biohealth Convergence, Seoul Women’s University, Seoul 01797, Republic of Korea; sunsj79@swu.ac.kr (S.S.); lucyeunji@swu.ac.kr (E.I.); 3 Department of Urology, Nowon Eulji Medical Center, Seoul 01830, Republic of Korea

**Keywords:** apoptosis, CLU, cFLIP, doxazosin, HSP27, prostate cancer

## Abstract

**Background:** This study investigated how the expression of heat shock protein 27 (HSP27), cellular FLICE-like inhibitory protein (cFLIP), and clusterin (CLU) affects the progression of cancer cells and their susceptibility to doxazosin-induced apoptosis. By silencing each of these genes individually, their effect on prostate cancer cell viability after doxazosin treatment was investigated. **Methods:** PC-3 prostate cancer cells were cultured and then subjected to gene silencing using siRNA targeting HSP27, cFLIP, and CLU, either individually, in pairs, or all together. Cells were then treated with doxazosin at various concentrations and their viability was assessed by MTT assay. **Results:** The study found that silencing the CLU gene in PC-3 cells significantly reduced cell viability after treatment with 25 µM doxazosin. In addition, the dual silencing of cFLIP and CLU decreased cell viability at 10 µM doxazosin. Notably, silencing all three genes of HSP27, cFLIP, CLU was most effective and reduced cell viability even at a lower doxazosin concentration of 1 µM. **Conclusions:** Taken together, these findings suggest that the simultaneous silencing of HSP27, cFLIP, and CLU genes may be a potential strategy to promote apoptosis in prostate cancer cells, which could inform future research on treatments for malignant prostate cancer.

## 1. Introduction

Prostate cancer is one of the most commonly diagnosed cancers in men worldwide and is responsible for a significant number of deaths. The early diagnosis of prostate cancer through prostate-specific antigen testing can lead to a successful treatment with surgery or radiation therapy. However, when prostate cancer progresses to castration-refractory prostate cancer (CRPC), it becomes difficult to treat with conventional hormone therapy or chemotherapy [1,2,3]. In these cases, α1-receptor blockers, such as doxazosin and terazosin, are widely used as drugs for the treatment of CRPC [4,5]. These drugs have a chemical structure known as a quinazoline nucleus, and many studies have suggested that they can induce apoptosis in prostate cancer cells by inhibiting anoikis and prostate cancer cell invasion [6,7,8].

Many signaling proteins are known to be involved in the apoptosis of prostate cancer cells. Heat shock protein 27 (HSP27) is a cell survival factor synthesized in response to stress, such as UV irradiation, oxidizing agents, inflammation, hypoxia, and tumors [9,10,11]. In the absence of stress, HSP27 plays an important role in maintaining cellular homeostasis as a molecular chaperone that folds, assembles, and transports polypeptides. However, HSP27 increases cell survival by inhibiting apoptosis by interfering with cytochrome c secretion from mitochondria, caspase 3 activity, and apoptosome formation [12,13,14]. In prostate cancer, HSP27 is associated with the pathologic stage, Gleason score, lymph node metastasis, shorter biochemical recurrence, and a poor clinical outcome [15,16].

Another protein involved in apoptosis regulation is the cellular FLICE-like inhibitory protein (cFLIP), which is expressed in three splice variants: cFLIPL, cFLIPS, and cFLIPR [17,18]. Each variant has different structures and properties, but they inhibit apoptosis by interacting with the adaptor protein FADD in downstream of the death receptors Fas, DE4, and DR5 [19]. The overexpression of cFLIP has been detected in various cancers, and apoptosis is induced when cFLIP expression is downregulated by various cytokines and drugs [17,20].

Clusterin (CLU) is a multifunctional glycoprotein complex found in body fluids and cells that is known to have two opposing actions as an apoptosis factor or a cell survival factor [21,22]. The overexpression of CLU protects cells from apoptosis induced by cellular stresses, such as chemotherapy, radiotherapy, or androgen/estrogen depletion [23,24]. On the other hand, CLU can also suppress survival genes in cancer cells, while increasing apoptosis [25,26]. For this reason, CLU is used as a marker of apoptosis undergoing in various benign and malignant tumor tissues [25,27].

Therefore, the aim of the study was to investigate whether the inhibition of the genes that are known to protect against cell death caused by anticancer drugs could enhance the efficacy of doxazosin-induced apoptosis in prostate cancer cells. To test this hypothesis, small interfering RNA (siRNA) technology was used to simultaneously knock out all three target genes in PC-3 prostate cancer cells. This approach was designed to determine whether silencing these genes would make the cancer cells more susceptible to apoptosis when treated with the drug doxazosin.

## 2. Materials and Methods

### 2.1. Cell Culture and Doxazosin Treatment

The human PC-3 cell line was obtained from the American Type Culture Collection (Manassas, VA, USA). The cells were maintained in DMEM purchased from Gibco BRL (Grand Island, NY, USA) and supplemented with 10% fetal bovine serum (Gibco BRL, Grand Island, NY, USA) and 1% penicillin/streptomycin (Gibco BRL, Grand Island, NY, USA). The cells were cultured in a humidified atmosphere at 37 °C with 5% CO_2_. When the cells reached 70% confluence, the culture medium was replaced with fresh serum-free medium, and then treated with 1, 10, and 25 µM doxazosin (Sigma-Aldrich, St. Louis, MO, USA). A serum-free medium containing 0.25% DMSO (Sigma-Aldrich, St. Louis, MO, USA) was used as the control.

### 2.2. MTT Assay

The 3-(4,5-dimethylthiazol-2-yl)-2,5-diphenyltetrazolium bromide (MTT) assay was used to evaluate cell viability. PC-3 cells were seeded at a density of 1.5 × 104 per well in 48-well plates and treated with 1, 10, and 25 μM of doxazosin. At 24, 48, and 72 h after treatment, 50 μL of the MTT (Sigma-Aldrich, St. Louis, MO, USA) solution was added to each well and incubated for 30 min. The supernatant was then removed and the MTT formazan was dissolved in 500 μL of DMSO. The plates were further incubated for 1 h, and the absorbance was measured at 595 nm using a spectrophotometer (Multiskan Ex, Thermo Fisher Scientific, Waltham, MA, USA).

### 2.3. Annexin-V Staining

Apoptosis was demonstrated using Annexin V-FITC/Propidium Iodide (PI) double staining. Cells were seeded in 8-well chamber slides, treated with doxazosin for 72 h, and then double-stained with Annexin V-FITC (Invitrogen, Carlsbad, CA, USA) and propidium iodide (Sigma-Aldrich, St. Louis, MO, USA). The cells cultured on the slides were washed with PBS and incubated in a binding buffer, including Annexin V-FITC and PI, for 20 min. After washing with distilled water, the nuclei were counterstained with Hoechst 33258 (Sigma-Aldrich, St. Louis, MO, USA). The cells were observed under a fluorescence microscope (TE-300, Nikon, Tokyo, Japan). The percentage of apoptosis was calculated by counting all cells at three locations in a high-magnification (×400) field of view and expressed as a percentage of the number of cells stained green with Annexin V-FITC.

### 2.4. siRNA Silencing of HSP27, cFLIP, and CLU Genes

To downregulate the expression levels of HSP27, cFLIP, and CLU mRNA in PC-3 cells, siRNA duplexes were transfected into PC-3 cells using Lipofectamine RNAiMAX (Invitrogen, Carlsbad, CA, USA). Cells were seeded in plates in the Opti-MEM medium (Gibco, Waltham, MA, USA) without penicillin/streptomycin. siRNA targeting HSP27 mRNA was purchased from Sigma (St. Louis, MO, USA), and cFLIP and CLU mRNA were purchased from Invitrogen (Carlsbad, CA, USA). The mRNA target sequences for HSP27 (Gene ID: 3315), cFLIP (Gene ID: 8837), and CLU (Gene ID: 1191) were designed using a siRNA template design tool (Ambion, Austin, TX, USA). A final concentration of 5 μM siRNAs was used with Lipofectamine RNAiMAX in the Opti-MEM media. After 24 h, the transfection medium was removed and the cells were replenished with a complete medium. The transfected cells were allowed to grow for 24, 48, and 72 h at 37 °C in a 5% CO_2_ incubator.

### 2.5. Total RNA Extraction and qRT-PCR

The cultured cells were homogenized with Trizol (Invitrogen, Carlsbad, CA, USA), according to the manufacturer’s instructions. The RNA was then extracted by precipitation with chloroform and isopropyl alcohol (Sigma-Aldrich, St. Louis, MO, USA), washed with 75% ethyl alcohol, and dissolved in RNase-DNase-free water (Invitrogen, Carlsbad, CA, USA). The concentration and purity of the extracted RNA were measured using a Nano-drop (Thermo Fisher Scientific, Waltham, MA, USA). Next, cDNA synthesis was performed in two steps: first, using the extracted RNA and oligo dT, and second, using dNTP (Takara Bio Inc., Shiga, Japan) and RTase (Invitrogen, Carlsbad, CA, USA) for double-strand synthesis in the RT buffer (Invitrogen, Carlsbad, CA, USA). qRT-PCR was then performed using the Light Cycler 480 real-time PCR system (Roche, Manheim, Germany) with a buffer solution containing template cDNA, each primer, and SYBR Green (Roche, Manheim, Germany).

### 2.6. Western Blot Analysis

Samples were homogenized in a lysis buffer. Equal amounts of proteins were separated by 12% SDS-PAGE and transferred to the PVDF membranes (Amersham; GE Healthcare, Buckinghamshire, UK). The membranes were incubated in a 3% casein-blocking solution (Komabiotech, Republic of Korea). The membranes were incubated with rabbit anti-cFLIP polyclonal antibody (H-202, Santa Cruz Biotechnology, Paso Robles, CA, USA), goat anti-HSP27 polyclonal antibody (C-20, Santa Cruz Biotechnology, Paso Robles, CA, USA), rabbit anti-clusterin polyclonal antibody (H-330, Santa Cruz Biotechnology, Paso Robles, CA, USA), and mouse anti-β-actin monoclonal antibody (H-2202, Santa Cruz Biotechnology, Paso Robles, CA, USA) at 4 °C overnight. The membrane was washed three times and incubated with donkey anti-rabbit IgG-HRP (Santa Cruz Biotechnology, Paso Robles, CA, USA) and donkey anti-mouse IgG-HRP (Santa Cruz Biotechnology, Paso Robles, CA, USA) at room temperature for 1 h each. The membrane was then washed and detected with an ECL Plus Western blot detection reagent (Amersham; GE Healthcare, Buckinghamshire, UK). The relative protein levels were determined using ScionImage (National Institutes of Health, Bethesda, MD, USA).

### 2.7. Statistical Analysis

The results are presented as the mean and the standard error of the mean (SEM). A Student’s *t*-test and one-way ANOVA with Tukey’s test were used for the data analysis. A *p*-value of less than 0.05 was considered statistically significant.

## 3. Results

### 3.1. Cell Viability and Apoptosis after Doxazosin Treatment

**Figure 1:**
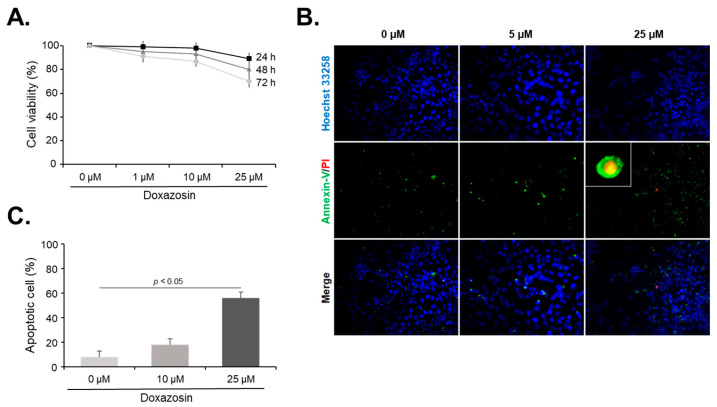
Effect of doxazosin on the cell viability and apoptosis of PC-3 cells. (**A**) Viability of PC-3 cells after doxazosin treatment. PC-3 cells were treated with 0, 10, or 25 µM doxazosin for 24 h. Cell viability assessed by MTT assay showed a significant decrease at 25 µM after 72 h compared to the control. (**B**) Detection of apoptotic cells after doxazosin treatment. Annexin-V (green fluorescence) revealed increased apoptosis correlating with doxazosin concentration. Conversely, necrotic cells identified by PI staining (red fluorescence) were rarely observed. Nuclei in cells were counter-stained with DAPI (blue fluorescence). Magnifications of ×200 and ×1000 (inset). (**C**) Percentage of apoptotic cells after doxazosin treatment. Apoptosis rates were significantly increased at 25 µM doxazosin. Data represent the mean ± SEM of three independent experiments performed in triplicate. Statistical significance (*p* < 0.05) was determined by a Student’s *t*-test for comparisons of 0 µM vs. 10 and 25 µM.

To examine the potential cytotoxic effect of doxazosin on PC-3 cells, we conducted an MTT assay to measure cell viability after treatment with 1, 10, and 25 µM doxazosin for 24, 48, and 72 h. The doxazosin treatment results in a dose- and time-dependent reduction in cell viability, with the greatest reduction observed in cells treated with 25 μM doxazosin for 72 h, which showed a 29.1 ± 6.5% reduction compared to the untreated controls (Figure 1A). To further elucidate the mechanism underlying this observed cytotoxic effect, we evaluated whether doxazosin-induced cell death was due to apoptosis or necrosis using Annexin-V and PI staining. Apoptotic cells, as evidenced by green fluorescence, were rarely detected in the doxazosin-untreated PC-3 cells, but increased in a dose-dependent manner after the doxazosin treatment. Conversely, only a negligible number of necrotic cells, as indicated by red fluorescence, were observed (Figure 1B). Importantly, we observed a significant increase in the rate of apoptosis with the 25 µM doxazosin (Figure 1C), suggesting that doxazosin-induced cytotoxicity in PC-3 cells is predominantly mediated by apoptosis rather than necrosis.

### 3.2. Expression of Survival-Related Genes after Doxazosin Treatment

**Figure 2:**
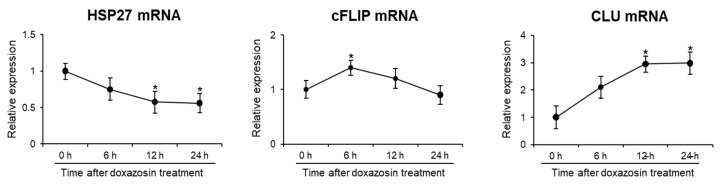
Gene expression of cell survival markers after doxazosin treatment in PC-3 cells. PC-3 cells were treated with 25 µM doxazosin for 6, 12, and 24 h. HSP27, cFLIP, and CLU mRNA levels were then quantified by qRT-PCR. HSP27 mRNA expression decreased progressively over 24 h, while cFLIP mRNA levels remained stable. In contrast, CLU mRNA expression showed a continuous increase. Data represent the mean ± SEM of three independent experiments, each conducted in triplicate. Statistical significance (* *p* < 0.05) was determined by a Student’s *t*-test for comparisons at 0 h vs. 6, 12, or 24 h.

To investigate the effect of doxazosin on the expression of HSP27, cFLIP, and CLU genes, the expression levels of each gene were measured by qRT-PCR after treatment with 25 µM doxazosin for 6, 12, and 24 h. The results show that the expression of HSP27 mRNA gradually decreased and reached its lowest level after 24 h of treatment. On the other hand, the expression of cFLIP mRNA exhibited a transient increase after 6 h of treatment, followed by a decrease from 12 h. In contrast, the expression of CLU mRNA increased steadily from 6 h after treatment and continued to increase until 24 h (Figure 2).

### 3.3. siRNA Efficiency of HSP27, cFLIP, and CLU Genes

**Figure 3:**
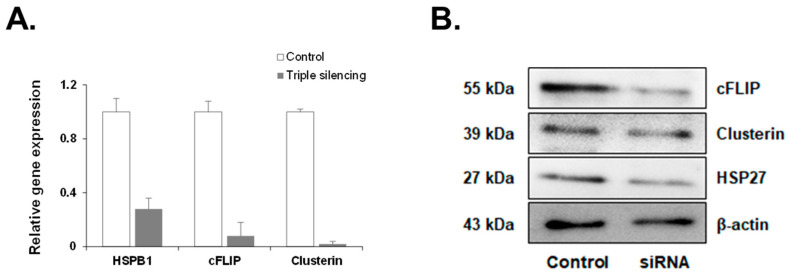
Gene and protein expressions after the siRNA silencing of HSP27, cFLIP, and CLU genes in PC-3 cells. The efficiency of siRNA knockdown was verified by analyzing mRNA and protein levels by qRT-PCR and Western blotting, respectively. (**A**) After the siRNA treatment, the mRNA expression of HSP27, cFLIP, and CLU was significantly reduced. Data represent the mean ± SEM of three independent experiments, each conducted in triplicate. (**B**) siRNA-treated cells show a corresponding decrease in the protein expression of these genes.

To assess the compatibility of siRNA targeting HSP27, cFLIP, and CLU genes in PC-3 cells, we conducted a qRT-PCR and Western blot analysis to detect the expression levels of each gene and protein. The expressions of HSP27, cFLIP, and CLU genes were significantly reduced after siRNA treatment for each gene (Figure 3A). In addition, the expression of all three protein types was reduced in the cells subjected to siRNA, similar to the qRT-PCR results (Figure 3B).

### 3.4. Cell Viability after siRNA of HSP27, cFLIP, and CLU Genes

**Figure 4:**
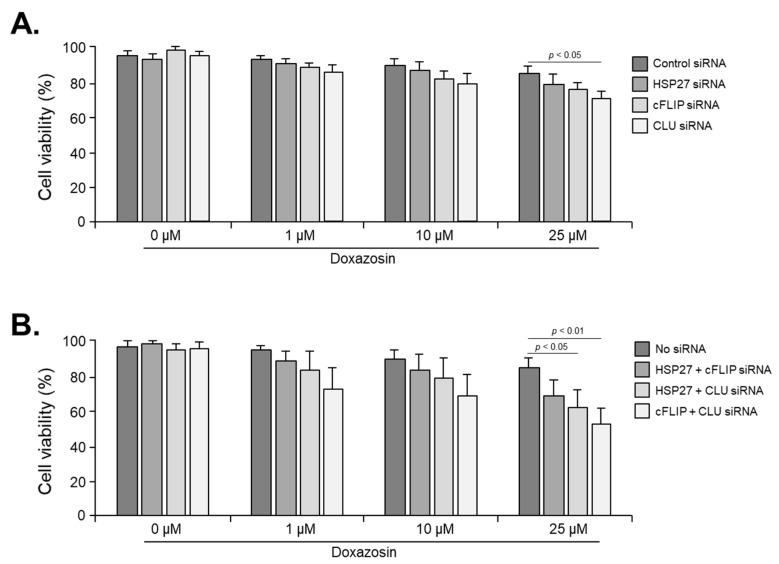
Effect of doxazosin on the viability of PC-3 cells subjected to single or dual siRNA silencing of HSP27, cFLIP, and CLU genes. PC-3 cells were treated with 0, 10, or 25 µM doxazosin for 24 h, and then cell viability was measured by the MTT assay. (**A**) Cell viability was significantly decreased at 25 µM doxazosin in cells with CLU gene silencing, while the silencing of HSP27 and cFLIP genes did not show this effect. (**B**) Dual siRNA silencing (HSP27 and cFLIP, HSP27 and CLU, or cFLIP and CLU) resulted in a significant decrease in cell viability at both 10 and 25 µM doxazosin. Data represent the mean ± SEM of three independent experiments, each conducted in triplicate. Statistical significance (*p* < 0.05) was determined by a one-way ANOVA and Tukey’s test followed by a Student’s *t*-test for 0 µM vs. 1, 10, and 25 µM comparisons.

**Figure 5:**
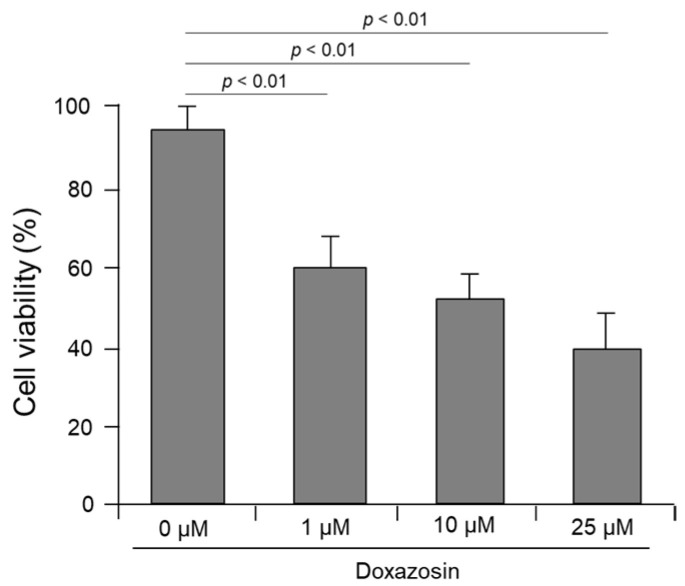
Effect of doxazosin on the viability of PC-3 cells subjected to triple siRNA silencing of HSP27, cFLIP, and CLU genes. Cell viability was significantly decreased at 10 and 25 μM doxazosin. In addition, even at 1 μM doxazosin, cell viability was significantly reduced compared to cells subjected to dual siRNA targeting two genes. Data represent the mean ± SEM of three independent experiments, each conducted in triplicate. Statistical significance (*p* < 0.05) was determined by a one-way ANOVA and Tukey’s test followed by a Student’s *t*-test for 0 µM vs. 1, 10, and 25 µM comparisons.

We evaluated the effect of doxazosin on the cell viability of PC-3 cells after siRNA targeting the HSP27, cFLIP, and CLU genes. In the control PC-3 cells not subjected to siRNA, cell viability was 92.4 ± 5.3%, 89.7 ± 4.8%, and 83.5 ± 6.3% after treatment with 1, 10, and 25 μM doxazosin, respectively, showing that there was no significant decrease (Figure 4A). In PC-3 cells subjected to single silencing targeting HSP27, cFLIP, and CLU genes, the overall cell viability was decreased after doxazosin treatment compared to the siRNA control group. Specifically, in cells subjected to siRNA targeting the CLU gene, cell viability was significantly decreased to 72.7 ± 5.1% after treatment with 25 μM doxazosin. However, in cells subjected to siRNA targeting HSP27 and cFLIP genes, cell viability was 80.4 ± 5.3% and 78.7 ± 4.8%, respectively, which did not show a significant decrease (Figure 4A). In cells subjected to dual silencing targeting HSP27 and cFLIP genes, cell viability was significantly decreased in all gene combinations compared to PC-3 cells subjected to single siRNA targeting one gene after doxazosin treatment. In cells subjected to dual silencing targeting HSP27 and CLU genes and cFLIP and CLU genes, cell viability was 62.6 ± 5.7 and 53.7 ± 6.9%, respectively, after 25 μM doxazosin treatment, indicating a significant decrease compared to the siRNA control group (Figure 4B). Finally, in PC-3 cells subjected to triple silencing of HSP27, cFLIP, and CLU genes, cell viability was significantly decreased at 10 and 25 μM doxazosin, with cell viability values of 52.1 ± 6.1 and 40.9 ± 7.1%, respectively. Even at 1 μM doxazosin, cell viability was significantly reduced to 58.3 ± 4.5% compared to the PC-3 cells subjected to dual siRNA targeting two genes (Figure 5).

## 4. Discussion

Doxazosin is a drug of the quinazoline α1-blocker class that has been shown to be a cell death inducer in both normal and cancerous prostate cells. Doxazosin has therefore been used extensively in the treatment of prostate cancer. However, the efficacy of doxazosin as a monotherapy is limited, prompting numerous efforts to improve its anticancer activity. As one of these efforts, we investigated whether the deletion of genes associated with cell survival, such as HSP27, cFLIP, and CLU, enhances the cell-death-inducing effect of doxazosin.

Before deleting those genes in PC-3 cells, we first confirmed the effects of doxazosin on cell viability and apoptosis using a prostate cancer cell line, PC-3 cells. Doxazosin induced a decrease in cell viability and an increase in apoptosis in a time- and dose-dependent manner in PC-3 cells after doxazosin treatment. These findings are consistent with many previously published research results. In a study using the DU-145 and PC-3 cell lines, doxazosin and terazosin were shown to induce apoptosis in the cells independently of alpha-adrenergic receptors and hormone receptors [28]. Another study reported that apoptosis occurred when doxazosin was exposed to normal prostate cells and PC-3 cells [29]. In addition, doxazosin is known to increase apoptosis by altering the mitochondrial membrane potential and increasing intracellular reactive oxygen species [30].

Next, we investigated whether HSP27, cFLIP, and CLU, as survival-related genes, were involved in the apoptosis induced by doxazosin. As a result of analyzing the expression of the three genes in PC-3 cells after doxazosin treatment, HSP27 mRNA expression decreased, while cFLIP mRNA expression temporarily increased before returning to normal levels. Conversely, CLU mRNA expression increased continuously after doxazosin treatment.

HSP27 is one of the well-known cell survival genes, and its expression decreases in cancer cells undergoing apoptosis [31]. HSP27 is a cell survival factor that is synthesized in response to cytotoxic stimuli and plays an important role in maintaining cell function in the absence of stress. Conversely, under stress, HSP27 inhibits apoptosis and increases cell survival by interfering with mitochondrial cytochrome c secretion and caspase-3 activation [32,33]. These results are not consistent with our results, showing that the expression of HSP27 mRNA is reduced by doxazosin treatment. However, it is proposed that HSP27 may not be involved in the doxazosin-induced apoptosis of prostate cells and that doxazosin may induce a decrease in HSP27 mRNA expression. Meanwhile, cFLIP is known as a cellular FLICE inhibitory protein that acts as an inhibitor of death receptor-mediated apoptosis. cFLIP inhibits apoptosis by interacting with the adapter protein FADD [34,35]. In addition, when FADD expression is downregulated by cFLIP silencing, drug-induced apoptosis is promoted [36,37]. In addition, CLU has been reported to be overexpressed to protect cells from cell death induced by cellular stress and extrinsic apoptotic signals [38]. CLU, also known as a testosterone-repressed prostate message-2 (TRPM-2), is reported to have a dual role as both an apoptosis factor and a cell survival factor [39]. Aberrant CLU expression has been associated with apoptosis in several cancers. It is also known that silencing the CLU gene in cancer cells reduces cell proliferation and increases apoptosis [40].

In this study, we showed that apoptosis in prostate cancer cells can be regulated by three genes important for cell survival, HSP27, cFLIP, and CLU. Apoptosis plays a critical role in the regulation of tissue homeostasis and is a key factor in the development and progression of cancer, including prostate cancer. Bcl-2 and Bax are two proteins that are centrally involved in the regulation of apoptosis, and their roles are particularly important in the context of prostate cancer. The overexpression of Bcl-2 has been frequently observed in prostate cancer. It has been reported that high levels of Bcl-2 are associated with the significant suppression of doxazosin-induced anoikis and cell invasion, which contributes to the survival and proliferation of prostate cancer cells [41]. Recent studies have reported that these cell survival genes, HSP27, cFLIP, and CLU, are associated with Bcl-2 and Bax expression. The inhibition of Hsp27 in DU145 and PC-3 prostate cancer cells not only reduced cell viability but also induced apoptosis by decreasing Bcl-2 levels and increasing Bax levels [42]. RU486 enhanced TRAIL-mediated apoptosis through the downregulation of Bcl-2 and cFLIP in human renal cell carcinoma Caki cells [43]. In addition, clusterin appears to contribute to an anti-apoptotic environment in prostate cancer cells, potentially influencing Bcl-2 activity and leading to increased cell survival, although the direct activation of Bcl-2 by clusterin has not been fully established [44]. Taken together, the reduction in HSP27, CFLIP, and CLU expression by doxazosin inhibits Bcl-2 function and thereby induces apoptosis in prostate cancer cells.

Considering these results, we hypothesized that the suppression of these genes could accelerate the apoptosis of PC-3 cells by doxazosin and conducted the small interfering RNA (siRNA) silencing of HSP27, cFLIP, and CLU genes in PC-3 cells. First, we examined cell viability after a single knockdown of these genes. We observed a significant decrease in cell viability in cells treated with CLU siRNA, but not in those treated with HSP27 and cFLIP siRNA. Our results show that HSP27 and cFLIP mRNA expression either decreased or remained unchanged, whereas CLU mRNA expression increased after doxazosin treatment. Considering these results, it seems that the sensitivity of doxazosin-induced apoptosis in PC-3 cells was enhanced by suppressing the expression of CLU mRNA, which is a survival factor upregulated by doxazosin treatment.

In this study, we also evaluated cell viability in PC-3 cells subjected to the dual silencing of HSP27, cFLIP, and CLU genes. Cell viability was significantly decreased in cells subjected to the dual silencing of cFLIP and CLU genes after doxazosin treatment compared with the control, but not in cells subjected to the dual silencing of HSP27 and cFLIP genes or HSP27 and CLU genes. These results suggest that HSP27 may interact with cFLIP and CLU, implying that silencing HSP27 gene expression compensates for the cell survival function of cFLIP and CLU genes. Next, we performed the triple silencing of the HSP27, cFLIP, and CLU genes to determine how the interactions of these genes affect the cell viability of PC-3 cells treated with doxazosin. As expected, sensitivity to doxazosin was increased in PC-3 cells subjected to the triple silencing of HSP27, cFLIP, and CLU genes, and cell viability was significantly reduced even in cells treated with 1 μM doxazosin compared to the control group. These results suggest that the triple silencing of HSP27, cFLIP, and CLU genes is likely to be more effective than single or dual silencing.

## 5. Conclusions

Our study shows that the administration of doxazosin after a single knockdown of the HSP27, cFLIP, and CLU genes in PC-3 cells, a prostate cancer cell line, leads to increased apoptosis. We observed that, when we applied dual siRNA to each of these genes, thereby increasing sensitivity to doxazosin, the cell survival rate after treatment was further reduced compared to the results with single siRNA. Notably, the simultaneous silencing of all three genes resulted in the lowest cell survival rate, suggesting that triple silencing may be more effective than single or dual silencing in inducing apoptosis in prostate cancer cells. This study is the first to perform the triple gene silencing of survival-related genes in PC-3 cells. We expect that these findings will provide fundamental data for future research in the treatment of aggressive forms of prostate cancer.

## Data Availability

The raw data supporting the conclusions of this article will be made available by the authors on request.
